# Type IIA topoisomerase (TOP2A) triggers epithelial-mesenchymal transition and facilitates HCC progression by regulating Snail expression

**DOI:** 10.1080/21655979.2021.2012069

**Published:** 2021-12-23

**Authors:** Yinying Dong, Xiangyin Sun, Kong Zhang, Xinjia He, Qian Zhang, Hao Song, Mingjin Xu, Haijun Lu, Ruimei Ren

**Affiliations:** a Department of Radiation Oncology, The Affiliated Hospital of Qingdao University, Qingdao,PR China; b Department of Intensive-care Unit, The Affiliated Hospital of Qingdao University, Qingdao, PR China; c Department of Gastroenterology, The Affiliated Hospital of Qingdao University, Qingdao,PR China

**Keywords:** TOP2A, HCC, EMT, Snail, prognosis

## Abstract

Type IIA topoisomerase (TOP2A) is upregulated in hepatocellular carcinoma (HCC) and its expression is positively correlated with poor prognosis. However, the underlying molecular mechanism of this connection are poorly understood. Hence, the present work aimed to examine the possible mechanisms which may be useful in identifying a potential therapeutic strategy. The differential expression of TOP2A mRNA in HCC as compared with adjacent normal tissue was analyzed using the Oncomine database. The expression levels of TOP2A in HCC specimens and cell lines were assessed by Western blot and RT-qPCR. Stable cell lines were generated to knockdown or overexpress TOP2A, and then cell growth, migration, and invasion were analyzed. Furthermore, this study examined epithelial-mesenchymal transition (EMT) as well as the activation of related pathways. Additionally, the correlation between TOP2A levels and E-cadherin/Snail expression was determined in 72 HCC specimens. Higher expression levels of TOP2A were observed in HCC in Oncomine datasets, and the results were verified using 40 pairs of HCC specimens and peritumoral tissues. TOP2A expression levels were remarkably elevated in cells with great metastatic capacity. In addition, HCC cell growth, migration, and invasion were suppressed after TOP2A knockdown in MHCC97H cells (MHCC97H-shRNA-TOP2A), while these capabilities were promoted in TOP2A-overexpressing Hep3B cells (Hep3B-TOP2A). Furthermore, EMT was inhibited in MHCC97H-shRNA-TOP2A cells, but induced in Hep3B-TOP2A cells. The induction of EMT by TOP2A was shown to be mediated by Snail, as TOP2A promoted Snail expression through the p-ERK1/2/p-SMAD2 signaling pathway. TOP2A level showed a negative correlation with E-cadherin, whereas a positive correlation with that of vimentin and Snail in human HCC specimens by immunohistochemistry analyses. Kaplan–Meier survival curves revealed that TOP2A upregulation showed a positive correlation with poor prognosis patients. Taken together, TOP2A possibly enhances the metastasis of HCC by promoting EMT through the mediation of the p-ERK1/2/p-SMAD2/Snail pathway. This indicates that TOP2A maybe a potential factor to predict the prognosis of HCC.

## Introduction

DNA topoisomerase family comprises type I and II topoisomerases, which induce double- and single-stranded breaks in DNA, respectively [1]. Based on differences in phylogenetic relationships and/or sequences of amino acids, these enzymes can further be subdivided into type IA, IB, IC, IIA, and IIB topoisomerases [2]. Type IIA topoisomerase (TOP2A) can relax the positive/negative DNA supercoils to regulate DNA topology, and solve chromatid separation and chromosome condensation in the processes of DNA transcription and replication [3–5]. In addition, TOP2A will accumulate in high levels in fast-dividing cells, besides, targets specific to TOP2A participate in cell growth [5]. An avalanche of evidence has shown that TOP2A is overexpressed in various cancers, including HCC, malignant peripheral nerve sheath tumor, hepatoblastoma, breast cancer (BC), esophageal cancer, gastroesophageal cancer, pancreatic cancer, colorectal cancer (CRC), and prostate cancer (PCa) [6–9]. The expression levels of TOP2A, a driver gene and a prognostic factor, predict the dismal prognosis of BC and HCC [7]. In prostate cancer, TOP2A expression has been demonstrated to be related to epigenetic modulation via the enhancer of zeste homolog2 (EZH2), while its abnormal expression is associated with the cancer phenotype [3,6]. Inhibition of TOP2A expression results in altered signaling: in the context of pancreatic cancer, TOP2A overexpression activates the β-catenin pathway [10], while TOP2A downregulation inhibits extracellular signal-regulated kinase (ERK) and AKT activity in colon cancer [11]. Based on the results of previous studies, TOP2A expression is possibly related to physiological activities within HCC, while its function(s) in HCC and the related pathways remain unclear.

This is the first study to investigate TOP2A with respect to its expression, significance in predicting HCC prognosis, and the underlying mechanism. To this end, we employed Oncomine databases, and Kaplan–Meier plotter (KM plotter) for analyzing the expression of TOP2A in HCC, the relationships between expression of TOP2A and prognosis of HCC patients. The roles of TOP2A in HCC cell proliferation, invasion, and EMT were also elucidated. Furthermore, we determined that the p-ERK1/2/p-SMAD2/Snail pathway mediates the TOP2A-induced EMT in HCC cells. In conclusion, the present work suggested that TOP2A can induce cell invasion and EMT in HCC cells; hence, it can be considered as a candidate therapeutic target.

## Materials and methods

### Data extraction

This study employed Oncomine data (http://www.oncomine.org) to analyze TOP2A levels in HCC patients. Information on TOP2A was explored by comparing the datasets of HCC and normal tissues [12].

### Selection and evaluation of patients

This work collected 112 HCC samples and 112 paired non-carcinoma hepatic samples. Among these samples, 40 pairs were snap-frozen, while 72 pairs were paraffin-embedded. Our study protocols were approved by the Research Ethics Committee of Fudan University Zhongshan Hospital (Shanghai, China) (Identification code: B2013-150).

### Cell lines and animals

This study applied the L02 healthy human liver cell line, together with several HCC cell lines (LM3, Hep3B, MHCC97H, Huh7). To be specific, the above cell lines were cultivated under 5% CO_2_ and 37°C conditions. In addition, the 4-6-week-old BALB/c male nude mice (Shanghai SLAC Laboratory Animal Co., Shanghai, China) were housed within the specific pathogen-free environment. The animal experimental protocols were approved by the Ethics Committee for Animal Experiments of the Qingdao University Animal Care Committee, Shandong, China (Permit Number: SYXK: 2008–0039).

### Chemicals, reagents, and kits

Inhibitors: LY3214996 (p-ERK1/2 inhibitor), SB203580 (p-P38 inhibitor), LY204002 (p-AKT inhibitor), and SP600125 (p-Jun N-terminal kinase (JNK) inhibitor) were obtained from Sigma-Aldrich (St.Louis, MO, USA).

### RNA extraction and RT-qPCR

This study conducted RT-qPCR according to previous report [13]. After extraction, we reverse-transcribed the extracted total RNA to prepare cDNA. For RT-qPCR, cDNA was amplified and detected with a Light Cycler 480 II (Roche, Mannheim, Germany). The 2^−ΔΔCt^ approach was employed to determine relative gene levels, with GAPDH being the endogenous reference. Supplementary Table 1 lists sequences for all the primers.

### Western blotting (WB) analysis

Total cellular or tissue proteins were isolated, separated through 10% SDS-PAGE, and transported on the PVDF membranes (Millipore, Boston, MA, USA). Thereafter, we incubated membranes using primary antibodies overnight under 4°C. Supplementary Table 2 presents all the antibodies utilized in this work. Thereafter, membranes were additionally incubated using the HRP-labeled secondary antibody (1:1000, Dingguo Bio, Beijing, China). Eventually, an electrochemiluminescence kit (Thermo, Waltham, MA, USA) was adopted for visualizing target bands [13].

### Immunohistochemistry (IHC) analyses

Tissue sections were rehydrated, then antigen retrieval and overnight incubation using specific primary antibodies under 4°Cwere performed. Later, the sections were further incubated using HRP-labeled secondary antibody (anti-rabbit 1:200; Dingguo Bio, Beijing, China) for a 30-min period at37°C. At last, 3,3ʹ-diaminobenzidine staining, as well as Mayer’s hematoxylin counter staining was performed. Photographs were later obtained from fourtypical sites by Leica QWin Plus v3 software (200× magnification) at identical setting parameters. In addition, Image-Pro Plus v6.2 software (Media Cybernetics Inc., Bethesda, MD, USA) was employed for measuring positive staining intensity. The section staining was examined under the same settings. Next, we determined the pooledoptical density values for the positive staining of every image, and later calculated the ratio of positive staining to the total area in every image [13]. Supplementary Table 2 lists the primary antibodies utilized in this work.

### Lentivirus construction and cell transfection

The pGCSIL-shRNA-TOP2A and pGC-FU-TOP2A cDNA lentiviral vectors were provided by Shanghai Genechem Company Co. Ltd. (Shanghai, China). With regard to knockdown TOP2A, its sequence was 5ʹ-ATCCTGCAG-GAATGGCATT-3ʹ. As a negative control, the sequence of non-silenced short hairpin RNA (shRNA) was 5ʹ-TTCTCCGAACGTGTCACGT-3ʹ. We also established the pcDNA3.1 vector that contained FLAG-tagged TOP2A, with pGC-FU lentiviral vector being the reference [13]. Later, we verified clones with stable transfection through RT-qPCR and WB assays.

### Cell Counting Kit-8 (CCK-8) proliferation assay

CCK-8 assay (Dojindo, Kumamoto, Japan) was conducted to determine HCC cell growth. The main steps areas follows. First, cell suspension (100 µL) was added into the culture plate wells (including 2000 cells). After cell adhesion, we added CCK-8 solution (10 µL) into all cells and incubated for 1 h in the culture incubator. Finally, we adopted a microplate reader for measuring cell proliferation at 450 nm. Wells with the CCK-8 solution and corresponding medium, with no cells added served as blank controls [13].

### Subcutaneous tumor formation in nude mice

Twelve male nude mice of SPF grade (no specific pathogen) 4–6 weeks old were purchased to adapt to the new environment for 1 week. The logarithmic growth cells were washed with serum-free medium for 2 times, and the single cell suspension was prepared. Each nude mouse was given a subcutaneous injection of 0.1 mL cell suspension that contained 1 × 10^7^ Hep3B cells or 5 × 10^6^MHCC97H cells in the upper left flank region. Tumor width and length at the site of inoculation were measured to evaluate tumor growth rate. At 10 days later, each tumor was dissected and fixed with 4% formaldehyde to analyze the pathology [13].

### In vivo assays for tumor metastasis

After the subcutaneous tumors grew to acertain size, they were cut tothe size of 1 × 1 × 1 mm^3^, before transplantation into the nude mouse livers (n = 6, each). After 8 weeks, euthanasia was performed. Pathological analysis was conducted to determine lung metastasis and intra-liver dissemination, while Image-Pro Plus software 6.0 (Media Cybernetics Inc., Silver Spring, MD, USA) was adopted for measuring fluorescence area according to previous description [13].

### Statistical analysis

Statistical analyses were completed by GraphPad Prism 5 v5.01 software (La Jolla, CA, USA). Differences in two groups were compared by Student’s t-test. One-way ANOVA as well as Post-Hoc Test (least significant difference, LSD) was adopted to compare data between two groups. Each assay was completed thrice independently and results were presented asmean ± SD. *p < 0.05, **p < 0.01, ***p < 0.001 represent the levels of statistical significance.

## Results

In this work, higher expression levels of TOP2A were observed in HCC in Oncomine datasets, and the results were verified using 40 pairs of HCC specimens and paracancerous tissues. We also found that TOP2A expression levels remarkably elevated in cells with great metastatic capacity. In addition, HCC cell growth, migration, and invasion were suppressed after TOP2A knockdown in MHCC97H cells (MHCC97H-shRNA-TOP2A), while these capabilities were promoted in TOP2A-overexpressing Hep3B cells (Hep3B-TOP2A). Furthermore, EMT was inhibited in MHCC97H-shRNA-TOP2A cells, but induced in Hep3B-TOP2A cells. The induction of EMT by TOP2A was shown to be mediated by Snail, as TOP2A promoted Snail expression through the p-ERK1/2/p-SMAD2 signaling pathway. TOP2A level showed a negative correlation with E-cadherin, whereas a positive correlation with that of vimentin and Snail in human HCC specimens. Kaplan–Meier survival curves revealed that TOP2Aupregulation showed a positive correlation with poor prognosis patients. Taken together, TOP2A possibly enhances the metastasis of HCC by promoting EMT through the mediation of the p-ERK1/2/p-SMAD2/Snail pathway.

### TOP2A expressions are upregulated in human HCC

**Figure 1.**
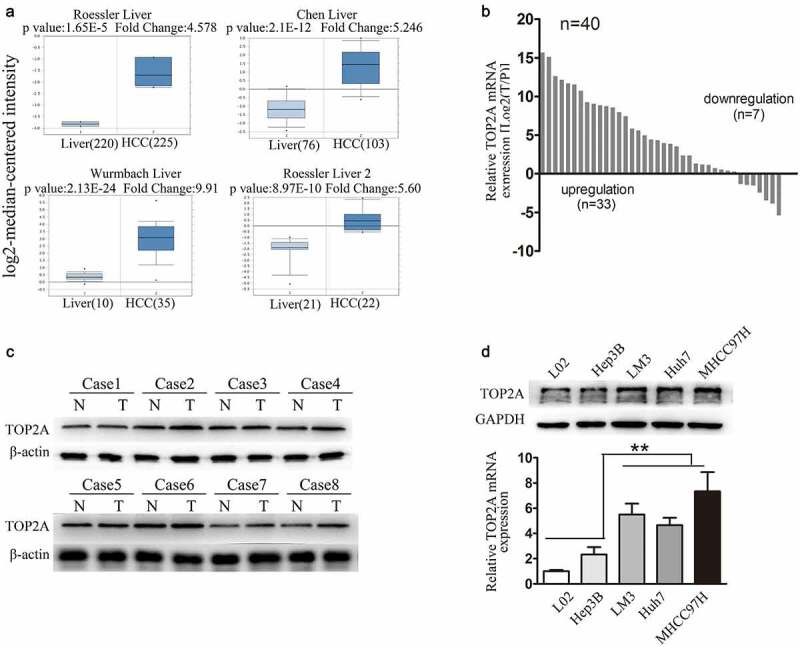
**TOP2A upregulated in HCC.**(a) Comparison of TOP2A mRNA levels between HCC and normal tissues in four research arrays via analysis of the Oncomine database. (b and c) Relative TOP2A expression levels in tumoral and peritumoral tissues were explored by RT-qPCR (b), Western blot (c). (d) Relative TOP2Aexpression in diverse HCC cells was determined through Western blot and RT-qPCR. Error bars indicate SD. *P < 0.05, **P < 0.01, ***P < 0.001

Four studies that measured TOP2A levels within HCC and matched non-carcinoma hepatic tissue samples were identified in the Oncomine database, with a total of 712 samples. The relative TOP2A levels in HCC as compared with hepatic tissues are shown in Figure 1(a). It indicates the increased TOP2A levels in HCC samples relative to the healthy hepatic samples. Subsequently, various approaches were used to show that TOP2A levels were increased in HCC. Firstly, RT-qPCR assays using a cohort of 40 specimens indicated that about 80% of the TOP2A mRNA levels within HCC tissues increased as compared with non-carcinoma samples (Figure 1(b)). This finding was validated by analyzing eight pairs of HCC samples from the cohort by Western blot analysis (Figure 1(c)). In addition, TOP2A levels within human HCC cells were analyzed. Relative to Hep3B cells with poor metastasis and healthy L02 liver cells, TOP2A protein and mRNA levels increased in cells with high metastasis, including Huh7, HCCLM3, and MHCC97H (Figure 1(d)).

### TOP2A induces HCC cell proliferation, invasion, and migration in vitro as well as lung metastasis in vivo

**Figure 2.**
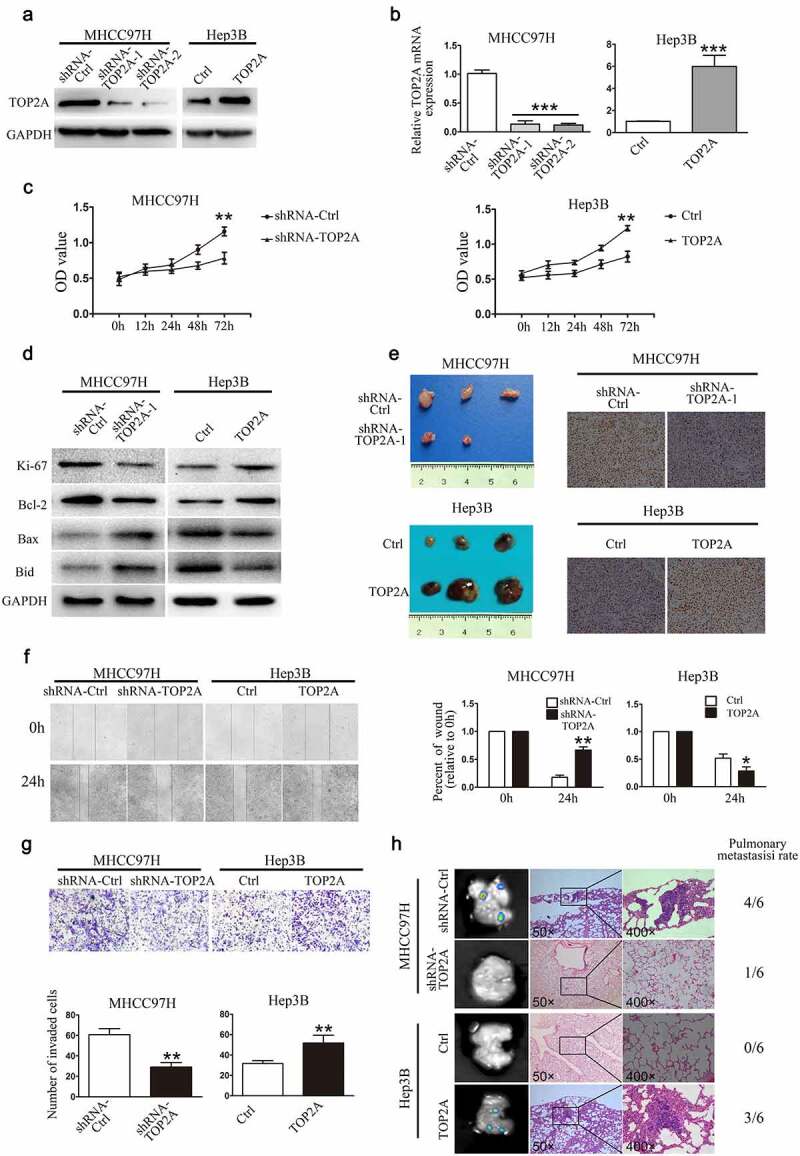
**TOP2A enhances HCC cell viability, invasion, and migration.** (a, b) Knockdown of TOP2A in MHCC97H cells and upregulation of TOP2Ain Hep3B cells were analyzed through Western blot (a) and RT-qPCR (b) (c) CCK-8 assay results showed that TOP2A enhanced the viability of MHCC97H and Hep3B cells. D. Apoptotic proteins were detected after changing TOP2A expression. E. Proliferation of MHCC97H cells and Hep3B cells after changing TOP2A expression was detected by subcutaneous tumor formation in nude mice; Ki-67 expression was also detected, Magnification, 400 × (f) The migration capacity was determined by scratch assays and by quantifying open area percentage. Magnification, 100 × . (g) Results of transwell Matrigel invasion assays following knockdown or overexpression of TOP2A in HCC cells are shown. Magnification, 200 × . (f) Lung tissues showing metastatic foci of MHCC97H-GFP and Hep3B -GFP. Typical images for lung tissue sections of every group and pulmonary metastasis rates are presented. Error bars indicate SD. *P < 0.05, **P < 0.01, ***P < 0.001

The effects of TOP2A knockdown in MHCC97H cells and stable overexpression in Hep3B cells were determined through RT-qPCR and Western blot (Figure 2(a-b)). Impact of TOP2A on MHCC97H and Hep3B cell viability was assessed using CCK8 assay, where knockdown of TOP2A decreased MHCC97H cell viability, while TOP2A overexpression in Hep3B cells promoted their proliferation (Figure 2(c)). Simultaneously, expressions of Ki-67 and anti-apoptotic Bcl-2 reduced while pro-apoptotic protein Bax, Bid, which act as key executors in apoptosis and play important roles in programmed cell death, were upregulated in MHCC97H-shRNA-TOP2A cells. On the contrary, Ki-67 and Bcl-2 induced in Hep3B-TOP2A cells relative to Hep3B-Ctrl cells, while the levels of Bax, Bid were downregulated (Figure 2(d)). Furthermore, for assessing the TOP2A role in HCC cell tumorigenicity, subcutaneous tumor formation experiments in nude mice were conducted. MHCC97H-shRNA-Ctrl and MHCC97H-shRNA-TOP2A groups were subcutaneously inoculated into male nude mice. As shown in Figure 2(e) and Figure S1 A, B, compared with the MHCC97H-shRNA-Ctrl cell group, the MHCC97H-shRNA-TOP2A cell group exhibited significantly reduced tumor volume, weight, and downregulation in Ki-67 expression. Accordingly, the Hep3B-TOP2A-generated xenograft size and weight, as well as Ki-67 expression remarkably increased relatively to Hep3B-ctrl-obtained xenografts. In wound closure assay, MHCC97H-shRNA-TOP2A cells displayed delayed wound closure as compared to that observed for MHCC97H-Ctrl cells. Moreover, TOP2A cDNA-transfected Hep3B (Hep3B-TOP2A) cells exhibited an increase in wound closure as compared to that observed for Hep3B-Ctrl cells (Figure 2(f)). In invasion assays, fewer invasive MHCC97H-shRNA-TOP2A cells were detected than that observed in MHCC97H-Ctrl cells, whereas there were more invasive Hep3B-TOP2A cells as compared with Hep3B-Ctrl cells (Figure 2(g)). After the subcutaneous tumors were implanted into the liver of nude mice, liver tumors were successfully established. As in Figure 2(h), the rate of lung metastases that occurred in the mice of MHCC97H-Ctrl group was 66.7% (4/6), whereas MHCC97H-shRNA-TOP2A group showed relatively less lung metastasis (16.7%, 1/6). In the Hep3B-TOP2A group, the pulmonary metastaticrate was 50% (3/6), and no mouse in the Hep3B-Ctrl group showed lung metastasis (0%, 0/6). In brief, TOP2A expression significantly promotes HCC metastasis.

### TOP2A induces EMT by promoting Snail expression

**Figure 3.**
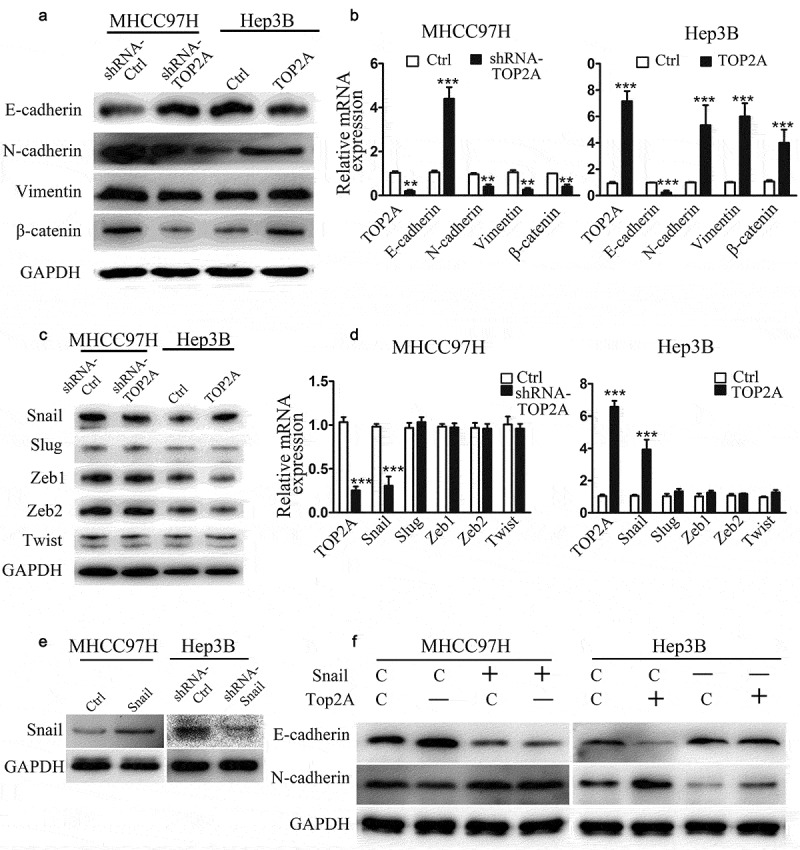
**TOP2A promotes EMT by upregulating Snail expression.** (a, b) Western blot (a) and RT-qPCR (b) results show the alterations of EMT marker levels after stable TOP2A downregulation and upregulation in HCC cells. (c,d) Role of TOP2A in Snail, Slug, Zeb1/2, and Twist levels was analyzed through Western blot (c) and RT-qPCR (d). (e) Western blot results showed upregulation of Snail in MHCC97H cells and downregulation of Snail in Hep3B cells. (f) N-cadherin and E-cadherin expressions in HCC cells after transfection with indicated vectors were assessed. Error bars indicate SD. *P < 0.05, **P < 0.01, ***P < 0.001

As TOP2A expression was observed to influence HCC cell proliferation, invasion, and migration, this study also examined the underlying mechanisms. HCC cell metastasis showed positive correlation with EMT, which was evidenced by induced expression of vimentin and N-cadherin (mesenchymal markers) and lost expression of E-cadherin (epithelial marker). Thus, we speculated that TOP2A may promote EMT in HCC cells. Through Western blot and RT-qPCR analyses, the expressions of EMT-associated proteins were observed to be significantly altered in response to the change in TOP2A expression. Following transfection with shRNA-TOP2A, E-cadherin level significantly elevated within MHCC97H cells in comparison with the control group, while N-cadherin, β-catenin, and vimentin exhibited reduced expressions. On the contrary, E-cadherin levels were reduced in Hep3B-TOP2A cells relative to Hep3B-Ctrl cells, while the levels of N-cadherin, β-catenin, and vimentin were elevated (Figure 3(a)). The mRNA levels of these markers in HCC cells were quantified by RT-qPCR (Figure 3(b)), with the results indicating that TOP2A is an inducer of EMT. Several key transcription factors (TFs), such as Twist, Slug, Snail, Zeb1/2, involved in EMT processes, are the factors necessary to transcriptionally suppress E-cadherin [4]. The Western blot and RT-qPCR results showed that unlike the trio of Slug, Twist, and Zeb1/2, Snail was positively correlated with TOP2A expression in MHCC97H and Hep3B cells (Figure 3(c-d)). In addition, the knockdown of TOP2A decreased the expression levels of Snail in MHCC97H cells, while Slug, Twist, and Zeb1/2 remained largely unchanged in expression levels. Meanwhile, Snail levels showed a significant increase when TOP2A was upregulated within Hep3B cells. To determine the role of Snail in TOP2A-induced EMT, Snail was upregulated in MHCC97H-shRNA-TOP2A cells, and downregulated in Hep3B-TOP2A cells. As in Figure 3(e-f), the inhibition of EMT was partially reversed in MHCC97H-shRNA-TOP2A cells. Moreover, the EMT induced by the overexpression of TOP2A was abolished after the transfection of shRNAs against Snail in Hep3B-TOP2A cells. Collectively, Snail has a key function in EMT of HCC cells as induced by TOP2A.

### TOP2A upregulates Snail expression via the p-ERK1/2-p-SMAD2(S425/250/255) signaling pathway

**Figure 4.**
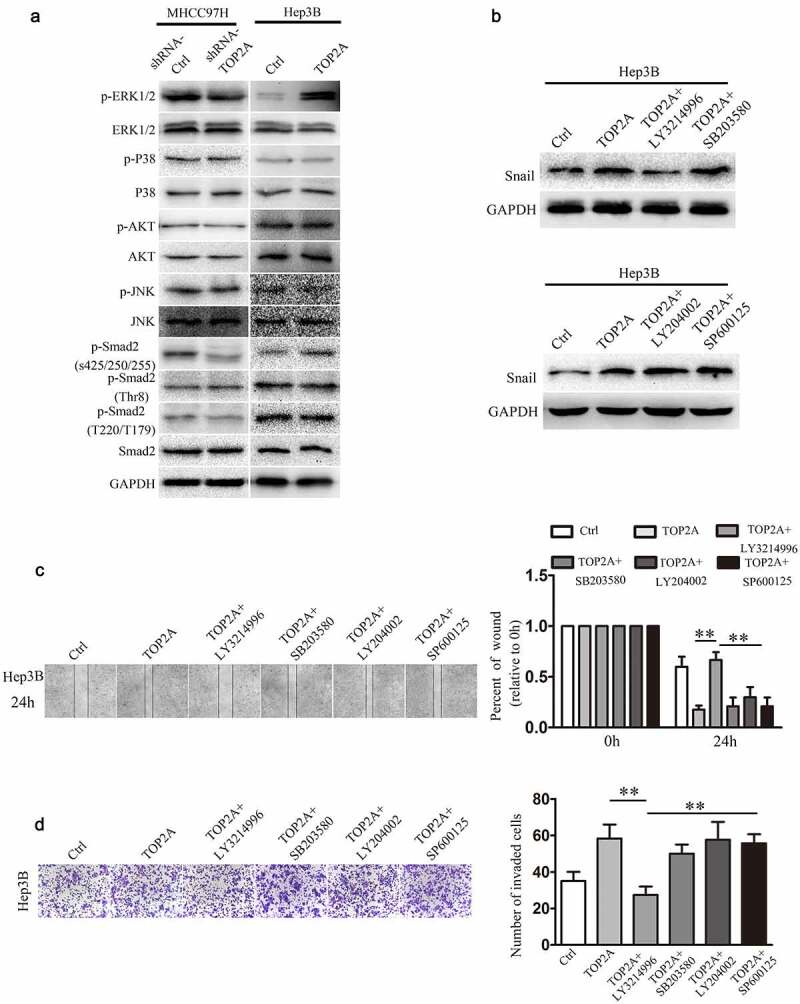
**TOP2A enhances Snail expression by regulating the p-ERK1/2/p-SMAD2(S425/250/255) signaling pathway.** (a) The activation of several signaling molecules was detected after knockdown or overexpression of TOP2A in HCC cells. (b) Hep3B cells after exposure to chemical inhibitors, including LY3214996 (ERK1/2), LY294002 (PI3K/AKT), SP600125 (SAPK/JNK), and SB203580 (P38) for a 60-min period. Snail expression in HCC cells was analyzed. (c,d) Migration and invasion capability were determined after exposure of Hep3B-TOP2A cells to chemical inhibitors. Error bars indicate SD. *P < 0.05, **P < 0.01, ***P < 0.001

To better elucidate the mechanisms of TOP2A in upregulating Snail expression, several proteins in signaling pathways, including ERK1/2, P38, AKT, JNK, and SMAD2, which are capable of altering the expression of Snail were investigated. As shown in Figure 4(a) and supplemental Figure 1(c), unlike P38, AKT, and JNK, the phosphorylation of ERK1/2 was remarkably downregulated after deletion of TOP2A in MHCC97H cells. Similarly, a markedly decreased phosphorylation of SMAD2 proteins, regulated by ERK1/2(S425/250/255), could be observed. In contrast, the levels of phosphorylated ERK1/2 and p-SMAD2(S425/250/255) were remarkably elevated when TOP2A was upregulated in Hep3B cells, whereas TOP2A made no difference to phosphorylation level of SMAD2 (Thr8) or SMAD2(T220/T179). To further demonstrate that TOP2A regulates Snail via ERK1/2, TOP2A-overexpressing Hep3B cells were treated with the inhibitors LY3214996 (50 μg/mL), SB203580 (5 μg/mL), LY204002 (50 μg/mL), and SP600125 (10 μg/mL). As shown in Figure 4(b), treatment with LY3214996 significantly decreased Snail expression, whereas the treatments with SB203580, LY204002, and SP600125 did not influence the expression of Snail. This study also analyzed the effects of these inhibitors in cell invasion and migration, and found out that only LY3214996 decreased the migration and invasion of Hep3B-TOP2A cells (Figure 4(c-d)). Taken together, the above findings suggest that phosphorylation of ERK1/2 mediates the TOP2A-induced overexpression of Snail.

### Correlation among TOP2A, E-cadherin, and Snail expression in HCC tissues

**Figure 5.**
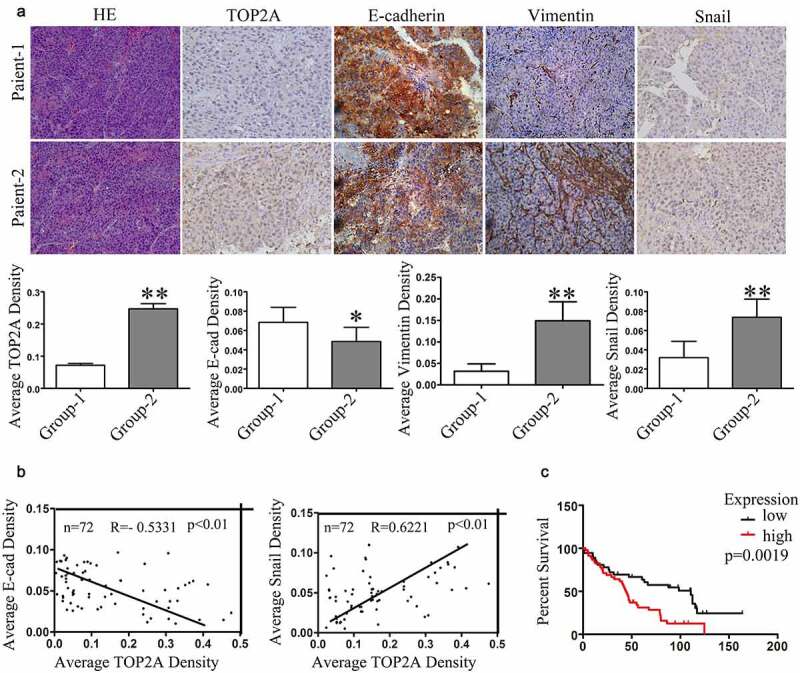
**Correlation in expression levels of TOP2A, E-cadherin, and Snail with patients’prognosis.** (a) Representative images of TOP2A, E-cadherin, Snail, and vimentin levels in HCC tissues analyzed through immunohistochemical staining, Magnification, 400 × . Column graph to quantitative analysis were provided. (b) Multiple linear regression revealed the negative correlation in TOP2A expression with E-cadherin level, while a positive correlation with Snail level. (c) Kaplan–Meier survival curve revealed that patients showing upregulation in TOP2A expression had remarkably poor prognosis as compared with those with TPO2A downregulation. Error bars indicate SD. *P < 0.05, **P < 0.01, ***P < 0.001

Seventy-two (72) HCC tumor samples were harvested for assessing the association of TOP2A expression with EMT in HCC cells through evaluation of TOP2A, E-cadherin, Snail, and vimentin levels. Representative pictures of immunohistochemical staining results are shown in Figure 5(a). In general, according to immunohistochemical analyses of the human HCC tissues, cases which showed TOP2A upregulation may have reduced expression of E-cadherin whereas along with increased expressions of vimentin and Snail (Figure 5(a)). According to multiple linear regression, TOP2A expression was in indirect proportion to E-cadherin level (R = −0.5331), but positively correlated with Snail (R = 0.6221) (Figure 5(b)). Collectively, the results further indicated that TOP2A promotes EMT in HCC cells. Moreover, based on Kaplan–Meier survival curves obtained using the Oncomine database, TOP2A upregulation showed a positive correlation with dismal patient’s survival (p = 0.0019; Figure 5(c), Supplementary Table 3). Thus, the above results suggested the potential of TOP2A as the efficient factor that predicts HCC prognosis.

## Discussion

TOP2A gene can encode one DNA topoisomerase responsible for controlling and altering DNA topology in the process of transcription [14]. In addition, TOP2A expressions in different cancers are suggested as the favorable prognostic biomarker that predict cancer progression and relapse, and it also serves as the risk factor for dismal survival [15–18]. In HCC, TOP2A has been shown to be upregulated in multiple studies. Wong (2009) [19] observed that high expression of TOP2A predicted microvascular invasion and advanced-stage tumor histology, which may indicate the early stage of HCC occurrence. Based on our results, TOP2A upregulation showed positive correlations with poor prognosis patients. Although TOP2A expression predicts the aggressive phenotype of cancers, the underlying molecular mechanisms have remained unreported.

Several studies have shown that TOP2A induces the progression of many cancers, such as HCC, breast, prostate, lung and colon cancer [6–9]. First, the expression of TOP2A in HCC were explored in the study. Using the Oncomine database, TOP2A was observed to be upregulated in cancers relative to healthy hepatic tissues. Furthermore, HCC tissues were collected to demonstrate that the expression levels of TOP2A were higher within HCC samples as compared with healthy non-carcinoma samples. The highly metastatic HCC cells exhibited increased expressions of TOP2A at protein and mRNA levels as compared with lowly metastatic and healthy cells. Additionally, other studies suggested that TOP2A induced proliferation and invasion of different tumors, such as lung cancer, PCa, and CRC [7–9]. According to in vivo/in vitro overexpression and depletion assays, TOP2A was found to have a critical effect in enhancing HCC invasion and migration, although the underlying mechanisms need to be further explored.

Overwhelming evidence suggested that EMT, which is regulated post-transcriptionally, is a reversible process that has an important effect on tumor development [20]. After EMT, cells with a typical mesenchymal morphology possess large intercellular spaces and loose connections [21]. In addition to changes in cell morphology, the occurrence of EMT can also be determined by analyzing changes in EMT-related marker protein levels [22]. After the onset of cellular EMT, the levels of epithelium-associated markers decrease, whereas those of mesenchymal-associated markers increase [23]. We speculated that EMT maybe related to HCC metastasis and migration induced by TOP2A. Interestingly, our results showed that TOP2A promoted EMT in HCC cells, which maybe the underlying key mechanism of this process. EMT can be induced by multiple transcription factors (TFs), such aszinc-finger binding TFs Snail1/Snail2 (called Snail/Slug as well) as well as some additional basic helix-loop-helix (bHLH) factors, like zinc-finger E-box-binding homeobox 1 (ZEB1)/ZEB2, or Twist [24]. Transcriptional repression mediated by TFs accounts for a frequently occurring mechanism related to dynamic CDH1 (E-cadherin encoding gene) silencing [25]. In this work, the expression of TOP2A promoted Snail levels, thus suppressing E-cadherin levels. Additional TFs remained unchanged by modulating the TOP2A levels in HCC cells. Furthermore, altering Snail expression could abolish the effects of TOP2A on EMT. Additionally, the expression levels of TOP2A showed a negative correlation with E-cadherin level, whereas a positive correlation with those of Snail in clinical HCC samples. Taken together, based on the above findings, TOP2A enhances the EMT in HCC cells; besides, Snail represents the key transcription factor that mediates this process.

The cell-signaling network underlying the TOP2A-mediated regulation of Snail is mostly unknown. TGF-β1/SMAD2 pathway has been well-recognized to promote EMT [26]. A variety of kinase pathways, like P38 mitogen activated protein kinase (MAPK), ERK, and c-JNK, are found to be independent of the SMAD2 signaling, which exerts their roles by regulating SMAD complexes [27–29]. Previous reports have observed the activation of PI3K/AKT signaling in EMT for multiple cell types [30,31]. Thus, we further investigated ERK1/2, P38, AKT, JNK, and SMAD2 for their phosphorylation and expression levels and observed that TOP2A can affect p-ERK1/2 and p-SMAD2 (S425/250/255) expression. Interestingly, when a P38 inhibitor (SB203580), ERK1/2 inhibitor (LY3214996), AKT inhibitor (LY204002), or JNK inhibitor (SP600125) was used to treat Hep3B cells overexpressing TOP2A, the TOP2A-induced Snail expression, migration, and invasion of the cells could only be suppressed by the ERK1/2 inhibitor (LY3214996). These results suggested that TOP2A may influence the expression of Snail by activating the pERK1/2/p-SMAD2 (S425/250/255) signaling pathway.

## Conclusions

In summary, we observed that TOP2A levels in HCC cells and tissues significantly increased, which may lead to the high incidence of distant metastasis and predicts dismal patient survival. Additionally, TOP2A was shown to potentially enhance HCC cell migration and invasion through triggering EMT, which is mediated by the p-ERK1/2/p-SMAD2 (S425/250/255)/Snail signaling pathway.

## Supplementary Material

Supplemental Material

## Highlights

- TOP2A is upregulated in human HCC; induces HCC cell growth, invasion, and migration in vitro and lung metastasis in vivo.
- TOP2A promotes HCC progression via EMT.
- The p-ERK1/2/p-SMAD2/Snail pathway mediates the TOP2A-induced EMT in HCC cells.

## Abbreviations

CCK-8, Cell Counting Kit-8; AKT, Protein Kinase B; EMT, epithelial-mesenchymal transition; GAPDH, glyceraldehyde phosphate dehydrogenase; ERK, extracellular signal-regulated kinase; HCC, hepatocellular carcinoma; MAPK, mitogen activated protein kinase; JNK, c-Jun N-terminal kinase; RT-qPCR, real-time quantitative polymerase chain reaction; TOP2A, Type II A topoisomerase; Zeb1, zinc-finger E-box binding homeobox 1.

## Declarations

**Ethics approval**: The use of human subjects was approved by the Research Ethics Committee of Fudan University Zhongshan Hospital (Shanghai, China) (Identification code: B2013-150). Each animal experiment was carried out under the approval by the Ethics Committee for Animal Experiments of the Qingdao University Animal Care Committee, Shandong, China (Permit Number: SYXK: 2008-0039).

## Data and Material Availability

All data utilized or analyzed in this work can be obtained from related authors upon request.
