# Supplementary material for: Type IIA topoisomerase (TOP2A) triggers epithelial-mesenchymal transition and facilitates HCC progression by regulating Snail expression
Source: Bioengineered. 2021 Dec 23;12(2):12967–79. doi: 10.1080/21655979.2021.2012069 (PMC8810028; doi:10.1080/21655979.2021.2012069)
Supplement: Supplemental Material [file KBIE_A_2012069_SM0179.zip › supplementary/supplemental table s1.docx]

| **sTable1.**Primer pairs used for qRT-PCR | |  |  |
| --- | --- | --- | --- |
| **Gene symbol** | **Sequence 5′-3′** | **NCBI accession number** | **length of PCR productions** |
| TOP2A  E-cadherin  N-cadherin  Vimentin  β-catenin  Snail  Twist  Slug  Zeb1  Zeb2  GAPDH | Forward: 5'- TGCACCCACTTGATTGAGACAT - 3'  Reverse: 5'- AGCCCTTAACCAGTACTTGCCT - 3'  Forward: 5'-AGCCCCGCCTTATGATTCTCTG - 3'  Reverse: 5'-TGCCCCATTCGTTCAAGTAGTCAT - 3'  Forward: 5'-GATGAAACGCCGGGATAAAGAAC - 3'  Reverse: 5'-GCTGCAGCTGGCTCAAGTCATAG - 3'  Forward: 5'-TTGAACGCAAAGTGGAATC - 3'  Reverse: 5'-AGGTCAGGCTTGGAAACA - 3'  Forward: 5'-TGACCTGACTGGGACTGAC - 3'  Reverse: 5'-AGGGCCTCGATTCGACTGCC - 3'  Forward: 5'-GACCCCAATCGGAAGCCTAACTAC - 3'  Reverse: 5'-AGCCTTTCCCACTGTCCTCATC - 3'  Forward: 5'-CGACGACAGCCTGAGCAAC - 3'  Reverse: 5'-CCACAGCCCGCAGACTTCTT - 3'  Forward: 5'-CCTCCATCTGACACCTCC - 3'  Reverse: 5'-CCCAGGCTCACATATTCC - 3'  Forward: 5'-AAGTGGCGGTAGATGGTA - 3'  Reverse: 5'-TTGTAGCGACTGGATTTT - 3'  Forward: 5'-TTCTGCGACATAAATACG - 3'  Reverse: 5'-GAGTGAAGCCTTGAGTGC - 3'  Forward: 5'- CTCCTCCACCTTTGACGC - 3'  Reverse: 5'- CCACCACCCTGTTGCTGT- 3' | NM_001067.4  Z13009.1  X57548.1  AH003024.2  X87838.1  NM_005985.4  Y10871.1  AF042001.1  NM_110794.2  NR_033258.2  Y18906.2 | 5,695 bp  4,778 bp  3,451 bp  5,359 bp  3,362 bp  1,705 bp  1,602 bp  4,034 bp  5,937 bp  2,736 bp  1076 bp |
| RT-qPCR quantitative real time reverse transcription polymerase chain reaction,  F, forward; R, reverse | |  |  |
