# Supplementary material for: Type IIA topoisomerase (TOP2A) triggers epithelial-mesenchymal transition and facilitates HCC progression by regulating Snail expression
Source: Bioengineered. 2021 Dec 23;12(2):12967–79. doi: 10.1080/21655979.2021.2012069 (PMC8810028; doi:10.1080/21655979.2021.2012069)
Supplement: Supplemental Material [file KBIE_A_2012069_SM0179.zip › supplementary/supplemental table s2.docx]

| **sTable 2.** Primary antibodies used in western blot and immunohistochemistry tests | | | |  |
| --- | --- | --- | --- | --- |
| **Antibody** | **Concentration(WB)** | **Concentration(IHC)** | **Company** | **catalogue number** |
| TOP2A  E-cadherin  N-cadherin  Vimentin  β-catenin  Snail  Twist  Slug  Zeb1  Zeb2  ERK1/2  p-ERK1/2  P38  p-P38  Smad2  p-Smad2(s425/250/255)  p-Smad2(Thr8)  p-Smad2(T220/T179)  Ki-67  Bcl-2  Bax  Bid  GAPDH | 1:1000  1:1000  1:1000  1:1000  1:1000  1:1000  1:1000  1:1000  1:1000  1:1000  1:2000  1:1000  1:1000  1:1000  1:1000  1:1000  1:1000  1:1000  1:1000  1:1000  1:800  1:1000  1:2000 | 1:150  1:200  /  1:200  /  1:150  /  /  /  /  /  /  /  /  /  /  /  /  1:100  /  /  /  / | Abcam  Abcam  Abcam  Cell Signaling Technology  Cell Signaling Technology  Abcam  Abcam  Cell Signaling Technology  Cell Signaling Technology  Cell Signaling Technology  Cell Signaling Technology  Abcam  Cell Signaling Technology  Abcam  Cell Signaling Technology  Abcam  Abcam  Abcam  Abcam  Abcam  Abcam  Abcam  Cell Signaling Technology | ab52853  ab40772  ab98952  46173  8840  ab180714  ab175430  9585  70512  97885  4376  Ab126445  8690  ab178867  5339  ab188334  ab254407  ab61066  ab16667  ab32124  ab32503  ab32060  5174 |
