# Supplementary material for: Type IIA topoisomerase (TOP2A) triggers epithelial-mesenchymal transition and facilitates HCC progression by regulating Snail expression
Source: Bioengineered. 2021 Dec 23;12(2):12967–79. doi: 10.1080/21655979.2021.2012069 (PMC8810028; doi:10.1080/21655979.2021.2012069)
Supplement: Supplemental Material [file KBIE_A_2012069_SM0179.zip › supplementary/supplemental table s3.docx]

**sTable 3.** Relationship between TOP2A expression and clinicopathological features

| **Clinicopathological features** | **Intratumoral expression of TOP2A** |
| --- | --- |
|  | **Negative no.** **Positive no.**  **P** |
| Age, years  ≤ 50  >50  Gender  Female  Male  HBsAg  +  −  HBeAg  +  −  HBV DNA, IU/mL  <5 × 10^2^  ≥ 5 × 10^2^  HCV  +  −  α -Fetoprotein, ng/ml  ≤ 20  >20  Liver cirrhosis  +  −  Tumor size, cm  ≤ 5  >5  Tumor number  Single  Multiple  Intrahepatic metastasis  +  −  Microvascular invasion  +  −  Tumor encapsulation  Complete  None  Recurrence site  No  Intrahepatic  Extrahepatic  Edmondson – Steiner grade  I – II  III – IV | 0.041  68 74  92 66  0.435  38 40  122 100  0.247  107 99  53 41  0.053  45 53  115 87  0.049  59 65  91 75  0.081  13 12  147 128  0.003  74 47  86 93  0.045  110 104  50 36  0.052  95 85  65 55  0.317  113 91  47 49  0.643  35 32  125 108  0.585  43 44  115 96  0.755  61 57  99 83  <0.001  82 43  52 72  22 21  0.387  105 94  55 46 |
