# Supplementary figures and images for: Type IIA topoisomerase (TOP2A) triggers epithelial-mesenchymal transition and facilitates HCC progression by regulating Snail expression
Source: Bioengineered. 2021 Dec 23;12(2):12967–79. doi: 10.1080/21655979.2021.2012069 (PMC8810028; doi:10.1080/21655979.2021.2012069)

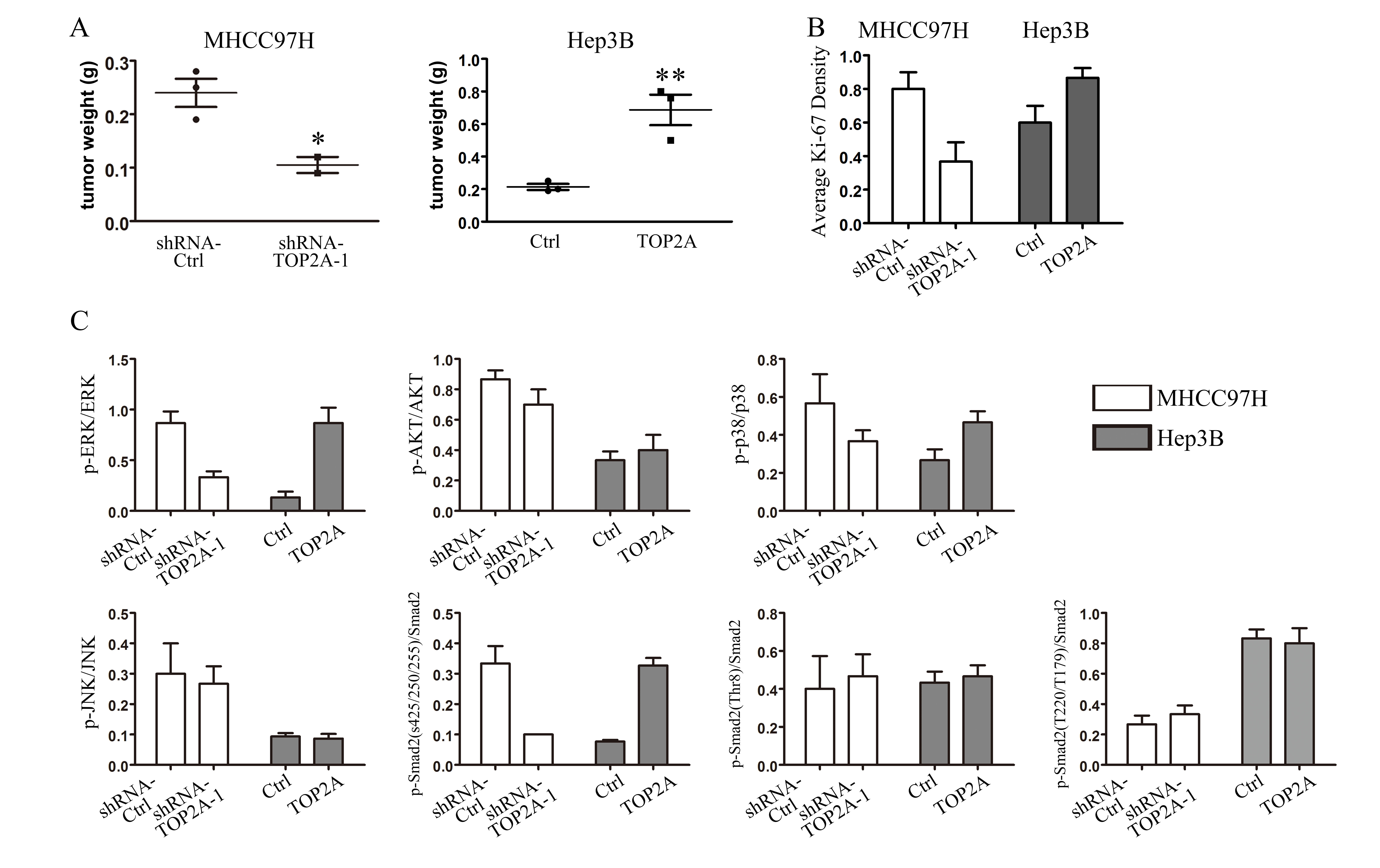

Supplement: Supplemental Material [file KBIE_A_2012069_SM0179.zip › supplementary/Supplemental figure 1.jpg]
